# The posterior tibial slope affects the measurement reliability regarding the radiographic parameter of the knee

**DOI:** 10.1186/s12891-024-07330-3

**Published:** 2024-03-07

**Authors:** Seung-Hun Lee, Je-Hyun Yoo, Dae-Kyung Kwak, Sung-Hwan Kim, Sung-Kuk Chae, Hyun-Soo Moon

**Affiliations:** 1 Department of Orthopedic Surgery, Hallym University Sacred Heart Hospital, Hallym University College of Medicine, Anyang, Republic of Korea; 2 Department of Orthopedic Surgery, Gangnam Severance Hospital, Yonsei University College of Medicine, Seoul, Republic of Korea

**Keywords:** Knee, Measurement reliability, Posterior tibial slope, Medial proximal tibial angle, Joint-line convergence angle

## Abstract

**Background:**

Posterior tibial slope (PTS) exhibits considerable variability among individuals and is anticipated to influence the accuracy of radiographic measurements related to the knee. Despite this potential impact, there is a lack of prior research investigating how PTS affects the accuracy of these measurements. Therefore, this study aimed to investigate the effect of PTS on the measurement reliability regarding the radiographic parameter of the knee.

**Methods:**

The medical records of patients who took full-length anteroposterior radiographs of the lower limb between January 2020 and June 2022 were evaluated retrospectively. Radiographic parameters related to the knee joint characteristics such as osteoarthritis grade, hip-knee-ankle angle, weight-bearing line ratio, medial proximal tibial angle (MPTA), lateral distal femoral angle, joint-line convergence angle (JLCA), and PTS were measured. Subjects were classified into 3 groups according to PTS (group A, PTS < 4°; group B, PTS ≥ 4° and < 8°; group C, PTS ≥ 8°), and the measurement reliability for the radiographic variables was compared between groups. The intra- and inter-observer agreements were assessed using the kappa coefficients, intra-class correlation coefficients (ICC), and Bland-Altman plots.

**Results:**

A total of 175 limbs (86 patients) were included in this study. As the intra- and inter-observer reliability for PTS ranged over 0.9, grouping was performed based on the average of the measured PTSs. The inter-observer reliability of the MPTA and JLCA decreased as the PTS increased (ICCs for MPTA in Groups A, B, and C: 0.889, 0.796, and 0.790, respectively; ICCs for JLCA in Groups A, B and C: 0.916, 0.859, and 0.843, respectively), whereas there were no remarkable differences in other variables. Similar trends were observed in the comparisons of intra-observer reliability and Bland-Altman plots also showed consistent results.

**Conclusion:**

The larger the PTS, the lower the measurement reliability regarding the radiographic parameters of the knee that includes the joint line, such as MPTA and JLCA. Given the occasional challenge in accurately identifying the knee joint line in patients with a relatively large PTS, careful measurement of radiographic parameters is crucial and utilizing repetitive measurements for verification may contribute to minimizing measurement errors.

**Supplementary Information:**

The online version contains supplementary material available at 10.1186/s12891-024-07330-3.

## Background

An evaluation of radiographic parameters in the management of knee disorders and injuries is essential for treatment planning and outcome assessment. The preoperative radiographic measurements in various knee surgeries, such as high tibial osteotomy and total knee arthroplasty, are critically important for planning the surgery, as they can significantly influence surgical outcomes. Similarly, postoperative radiographic measurements play a crucial role in the assessment following surgery and can substantially impact the postoperative management of patients. In particular, radiographic assessments in the coronal plane, i.e., measurements on anteroposterior (AP) images, are the most prominently utilized in clinical practice. This applies not only to osteoarthritis, but also encompasses cases involving fractures, cartilage lesions, ligament injuries, and meniscus tears [1–8]. Given their practical significance, ensuring the accuracy and reproducibility of radiographic measurements is imperative.

However, achieving consistent accuracy in the measurement of radiographic parameters is not always feasible. The knee joint, with its relatively complex osseous structure compared to other joints, exhibits substantial individual morphological variability [9–11]. These factors can contribute to the challenge of precise measurements. In particular, these characteristics become more evident in the proximal tibial surface. The morphology of the proximal tibial surface differs between the medial and lateral compartments of the knee, with noteworthy inter-individual differences [12–17]. Furthermore, as it directly mirrors the articular surface, its variability potentially affects the measurement outcomes of radiographic variables that include the joint line. Hence, the posterior tibial slope (PTS), indicating a posterior inclination of the tibial articular surface, is anticipated to influence the measurement reliability of radiographic parameters of the knee. The radiographic appearance in the sagittal plane is presumed to be mirrored to some extent in the coronal plane. Accordingly, evaluating the characteristics of radiographic variables in AP images, including their measurement reliabilities, can be contemplated through variables reflecting the features of the proximal tibial surface in the sagittal plane. Nevertheless, there is currently a gap in the existing research as no studies have been undertaken to investigate this issue.

Therefore, this study aimed to investigate the effect of PTS on the measurement reliability regarding the radiographic parameter of the knee. It was hypothesized that a larger PTS results in lower measurement accuracy for radiographic parameters involving the knee joint line.

## Methods

### Patient recruitment

**Fig. 1:**
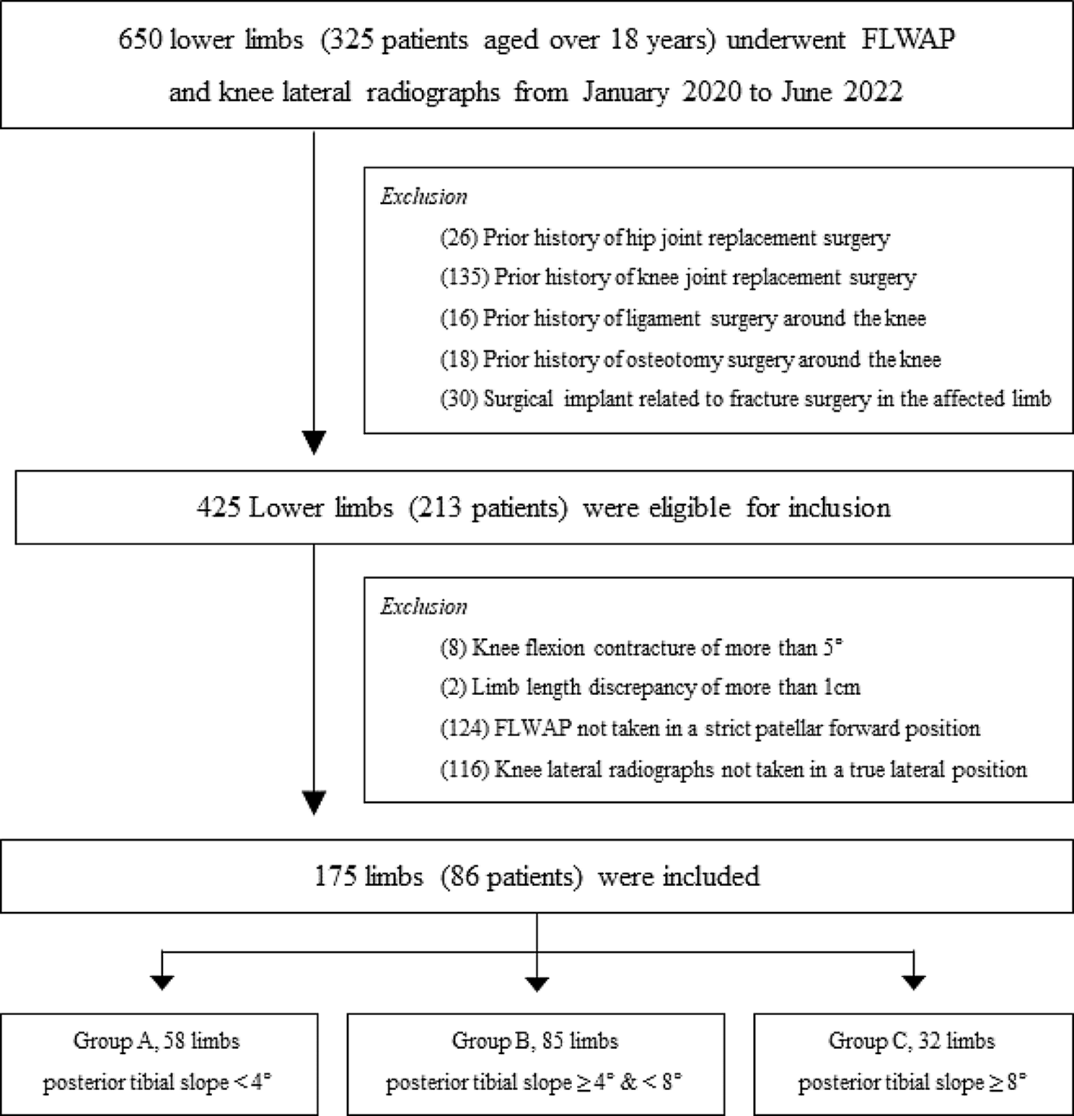
Flowchart of patient inclusion in the study *FLWAP*, full-length weight-bearing anteroposterior radiograph

This study obtained approval from the institutional review board of Hallym University Sacred Heart Hospital (approval number: 2022-12-015), and the retrospective nature of the study led to a waiver of the informed consent requirement. Electronic medical records of patients who had undergone full-length weight-bearing anteroposterior (FLWAP) radiographs and knee lateral radiographs at our institution between January 2020 and June 2022 were subjected to retrospective review. Patients were excluded based on the following criteria: (1) age of 18 years or younger; (2) prior hip/knee joint replacement surgery; (3) prior ligament surgery around the knee; (4) prior osteotomy surgery around the knee; (5) presence of surgical implant around the knee. Additionally, among eligible patients, those who met the following conditions that could potentially influence measurement accuracy were additionally excluded to ensure more precise measurements [18]: (1) knee flexion contracture > 5°; (2) limb length discrepancy of over 1 cm; (3) FLWAP radiographs not captured in a strictly patellar forward position; (4) knee lateral radiographs not taken in a true lateral position. Consequently, the patients in this study were classified into three groups according to their PTS measurements: Group A included limbs with PTS < 4°, Group B included limbs with PTS ≥ 4° and < 8°, and Group C included limbs with PTS ≥ 8° (Fig. 1).

### Demographic data and radiograph acquisition

The baseline demographic data included an assessment of age, gender, side, and the correspondence with the affected limb. The affected limb referred to the lower extremity designated for examination or intervention. All patients admitted to our institution due to knee pain underwent FLWAP radiographs, posteroanterior (PA) radiographs of the knee, and knee lateral radiographs to evaluate the characteristics of the knee joint and establish treatment strategies.

For FLWAP radiographs, images were captured with the patella directed towards the X-ray source, while maintaining a focus-to-film distance of 300 cm (Innovision-SH, Shimadzu, Japan; GC85A, Samsung Electronics, Korea). These images were automatically merged to form a composite image. PA radiographs of the knee were taken at about 30° of knee flexion, known as the schuss view [19]. FLWAP and knee PA radiographs were omitted for patients unable to bear weight due to pain or discomfort. Knee lateral radiographs were obtained with complete overlapping of both femoral condyles, achieved at approximately 30° of knee flexion.

### Radiographic measurements

**Fig. 2:**
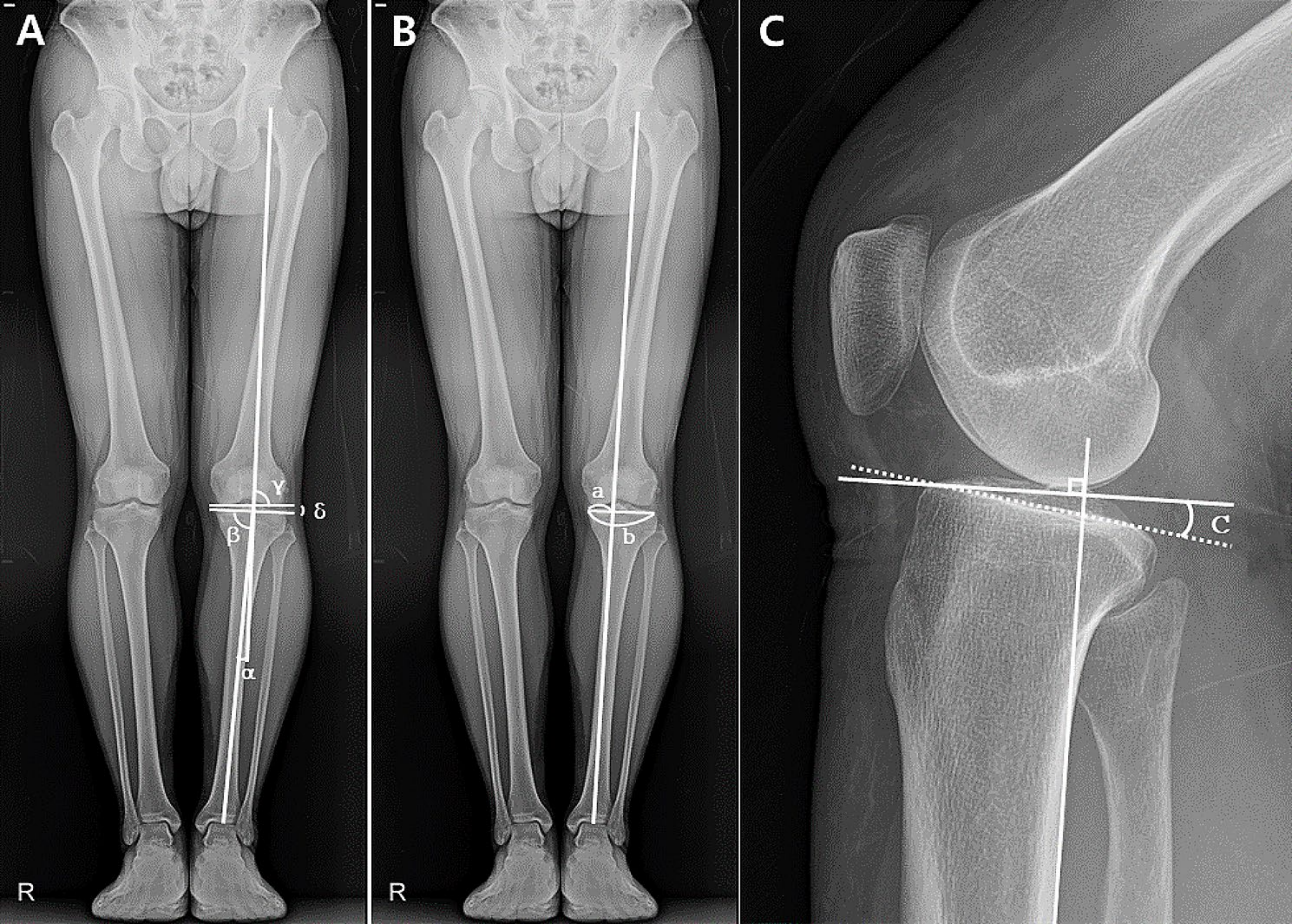
(**A**) Radiographic measurements of hip-knee-ankle angle (α, °), medial proximal tibial angle (β, °), lateral distal femoral angle (γ, °), and joint-line convergence angle (δ, °). (**B**) Radiographic measurement of weight-bearing line ratio (a/b x 100, %). (**C**) Radiographic measurement of posterior tibial slope (c, °)

Various radiographic parameters reflecting the characteristics of the knee joint were utilized in the evaluation, which include osteoarthritis grade, hip-knee-ankle (HKA) angle, weight-bearing line ratio (WBLR), medial proximal tibial angle (MPTA), lateral distal femoral angle (LDFA), joint-line convergence angle (JLCA), and PTS (Fig. 2). The radiographic osteoarthritis grade was assessed in both FLWAP and knee PA radiographs, according to the Kellgren-Lawrence grading system [20]. HKA angle, WBLR, MPTA, LDFA, and JLCA were measured using FLWAP radiographs [1, 21, 22]. The HKA angle was measured as the angle formed between the mechanical axis of the femur and the mechanical axis of the tibia [21]. The WBLR was determined by measuring the distance from the medial edge of the proximal tibia to the point where the weight-bearing line intersects the articular surface of the proximal tibia; this measurement is then divided by the overall width of the tibial articular surface [23]. MPTA was defined as the angle between the tibial mechanical axis and the line tangent to the articular surface of the proximal tibia [14]. LDFA was measured as the angle between the femoral mechanical axis and the line tangent to the articular surface of the distal femur [24]. JLCA was determined as the angle formed between the line tangential to the distal femur and the tibial plateau [25]. The PTS was measured on knee lateral radiographs and defined as the angle formed by a line tangent to the medial tibial plateau and the posterior cortical line of the proximal tibia [26]. Furthermore, patellar rotation relative to the femoral condyle was examined to confirm whether the FLWAP radiograph was taken in a patellar forward position [18]. A patellar rotation of 5% or less was determined to correspond to a strict patellar forward position. In addition, limb length discrepancy was evaluated by comparing the lengths of both lower limbs, following the methods described by Lang et al. [27].

Radiographic measurements were performed using a picture archiving and communication system (INFINITT M6 6062 workstation, INFINITT Healthcare Co. Ltd., Korea). The measurements were performed by two orthopedic surgeons who were blinded to patient information, as well as to each other’s data and their own previously recorded measurements. The two observers are orthopedic surgeons specializing in knees and have extensive experience in measuring alignment parameters. To minimize assessment bias, both observers took measurements at four-week intervals.

### Statistical analysis

The statistical analysis was conducted with IBM SPSS Statistics for Windows (version 26.0; Armonk, NY, USA). For statistical analysis of continuous variables, the mean values of measurements from both observers were employed. For categorical variables, individual measurement values from both observers were used, as they cannot be presented as average values. Following the measurements, the assessment of intra- and inter-observer reliabilities was conducted. Intra-class correlation coefficients (ICC) with 95% confidence intervals, utilizing a two-way random effects model, were calculated for continuous variables. Weighted kappa coefficients were employed to assess the measurement reliability for categorical variables. Furthermore, to analyze bias and the limits of agreement for continuous variables, Bland-Altman plots were derived. A *P* value of < 0.05 was considered statistically significant.

## Results

A total of 175 limbs (86 patients) were included in this study. The baseline characteristics and radiographic data of the subjects are summarized in Table 1. As the intra- and inter-observer reliabilities for PTS consistently exceeded 0.9 (Supplementary Material 1), grouping was performed based on the average of the measured PTS values, resulting in 58 limbs for Group A, 85 for Group B, and 32 for Group C (Fig. 1).

**Table 1:** Baseline demographic data and radiographic parameters

Variables	Overall subjects (*n* = 175)
Demographic data	
Age, year	59.95 ± 13.01
Sex	
Male/ Female	63/ 112
Side	
Right/ Left	86/ 89
Affected limb	
Yes/ No	104/ 71
Radiographic parameters (observer 1)	
Hip-knee-ankle angle, °^a^	4.66 ± 4.08
Weight-bearing line ratio, % ^a^	27.03 ± 18.27
Medial proximal tibial angle, ° ^a^	85.00 ± 2.43
Lateral distal femoral angle, ° ^a^	88.40 ± 2.08
Joint-line convergence angle, ° ^a^	1.46 ± 2.24
Posterior tibial slope, ° ^a^	5.52 ± 2.89
K-L grade (FLWAP radiographs)	
0/ 1/ 2/ 3/ 4 (Measurement 1)	56/ 47/ 31/ 23/ 18
0/ 1/ 2/ 3/ 4 (Measurement 2)	65/ 40/ 29/ 25/ 16
K-L grade (PA radiographs)	
0/ 1/ 2/ 3/ 4 (Measurement 1)	27/ 58/ 35/ 23/ 32
0/ 1/ 2/ 3/ 4 (Measurement 2)	37/ 51/ 32/ 22/ 33
Radiographic parameters (observer 2)	
Hip-knee-ankle angle, ° ^a^	4.99 ± 4.38
Weight-bearing line ratio, % ^a^	28.51 ± 17.59
Medial proximal tibial angle, ° ^a^	85.43 ± 2.48
Lateral distal femoral angle, ° ^a^	87.90 ± 2.09
Joint-line convergence angle, ° ^a^	1.88 ± 2.25
Posterior tibial slope, ° ^a^	5.55 ± 2.85
K-L grade (FLWAP radiographs)	
0/ 1/ 2/ 3/ 4 (Measurement 1)	39/ 60/ 33/ 29/ 14
0/ 1/ 2/ 3/ 4 (Measurement 2)	41/ 60/ 32/ 29/ 13
K-L grade (PA radiographs)	
0/ 1/ 2/ 3/ 4 (Measurement 1)	31/66/25/26/27
0/ 1/ 2/ 3/ 4 (Measurement 2)	31/70/19/30/25

*K-L* Kellgren-Lawrence, *FLWAP* full-length weight-bearing anteroposterior, *PA* posteroanterior

^a^ The average of Measurement 1 and Measurement 2

**Fig. 3:**
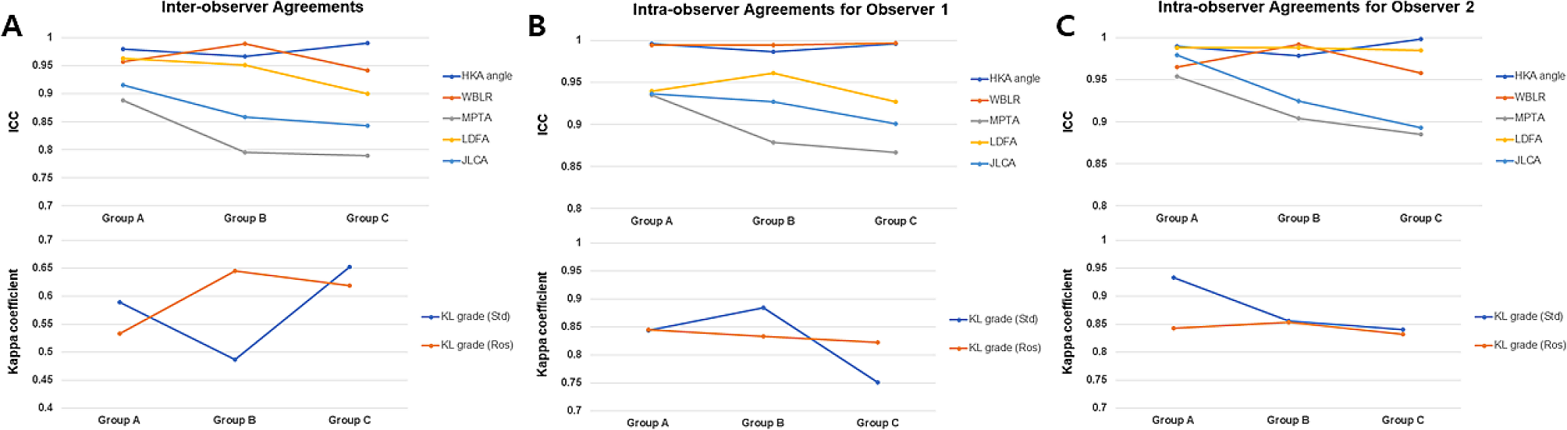
(**A**) Inter-observer measurement agreement between observer 1 and observer 2. (**B**) Intra-observer measurement agreement in observer 1. (**C**) Intra-observer measurement agreement in observer 2

In the comparison of inter-observer reliabilities among the 3 groups, the measurement reliabilities for MPTA and JLCA were found to be lower in Group B than in Group A, and further reduced in Group C than in Group B (ICCs for MPTA in Groups A, B, and C: 0.889, 0.796, and 0.790, respectively; ICCs for JLCA in Groups A, B and C: 0.916, 0.859, and 0.843, respectively) (Table 2) (Fig. 3). Whereas, no significant associations were identified between other radiographic variables and measurement reliabilities. These findings remained consistent in the intra-observer measurement reliabilities. This held for both Observer 1 (ICCs for MPTA in Groups A, B, and C: 0.935, 0.879, and 0.867, respectively; ICCs for JLCA in Groups A, B and C: 0.937, 0.927, and 0.901, respectively) and Observer 2 (ICCs for MPTA in Groups A, B, and C: 0.954, 0.904, and 0.885, respectively; ICCs for JLCA in Groups A, B and C: 0.980, 0.925, and 0.893, respectively) (Table 3) (Fig. 3). Aforementioned findings indicate that as PTS increases, the measurement reliability for MPTA and JLCA variables decreases. Bland-Altman analyses also revealed that, as PTS increased, the 95% limits of agreement between measured values expanded, consistently observed in both intra- and inter-observer comparisons (Supplementary Material 2, 3, and 4).

**Table 2:** Inter-observer measurement agreements between observer 1 and observer 2

	ICC	95% CI	*p*-value	Kappa value
Group A (*n* = 58)				
Hip-knee-ankle angle	0.98	0.967–0.988	< 0.001	
Weight-bearing line ratio	0.958	0.927–0.976	< 0.001	
Medial proximal tibial angle	0.889	0.766–0.942	< 0.001	
Lateral distal femoral angle	0.963	0.858–0.985	< 0.001	
Joint-line convergence angle	0.916	0.765–0.961	< 0.001	
K-L grade (FLWAP radiographs)				0.589
K-L grade (PA radiographs)				0.533
Group B (*n* = 85)				
Hip-knee-ankle angle	0.967	0.949–0.979	< 0.001	
Weight-bearing line ratio	0.99	0.985–0.994	< 0.001	
Medial proximal tibial angle	0.796	0.679–0.870	< 0.001	
Lateral distal femoral angle	0.951	0.901–0.973	< 0.001	
Joint-line convergence angle	0.859	0.780–0.909	< 0.001	
K-L grade (FLWAP radiographs)				0.487
K-L grade (PA radiographs)				0.645
Group C (*n* = 32)				
Hip-knee-ankle angle	0.991	0.949–0.997	< 0.001	
Weight-bearing line ratio	0.942	0.879–0.972	< 0.001	
Medial proximal tibial angle	0.79	0.569–0.898	< 0.001	
Lateral distal femoral angle	0.9	0.491–0.966	< 0.001	
Joint-line convergence angle	0.843	0.681–0.923	< 0.001	
K-L grade (FLWAP radiographs)				0.652
K-L grade (PA radiographs)				0.619

*ICC* Intra-class correlation coefficients, *CI* Confidence interval, *K-L* Kellgren-Lawrence, *FLWAP* full-length weight-bearing anteroposterior, *PA* posteroanterior

**Table 3:** Intra-observer measurement agreements

	Intra-observer measurement agreements in observer 1				Intra-observer measurement agreements in observer 2			
	ICC	95% CI	*p*-value	Kappa value	ICC	95% CI	*p*-value	Kappa value
Group A (*n* = 58)								
Hip-knee-ankle angle	0.996	0.993–0.997	< 0.001		0.990	0.983–0.994	< 0.001	
Weight-bearing line ratio	0.995	0.991–0.997	< 0.001		0.965	0.942–0.980	< 0.001	
Medial proximal tibial angle	0.935	0.890–0.962	< 0.001		0.954	0.923–0.973	< 0.001	
Lateral distal femoral angle	0.940	0.899–0.964	< 0.001		0.988	0.981–0.993	< 0.001	
Joint-line convergence angle	0.937	0.894–0.963	< 0.001		0.980	0.967–0.988	< 0.001	
K-L grade (FLWAP radiographs)				0.844				0.933
K-L grade (PA radiographs)				0.845				0.843
Group B (*n* = 85)								
Hip-knee-ankle angle	0.987	0.980–0.991	< 0.001		0.979	0.968–0.986	< 0.001	
Weight-bearing line ratio	0.995	0.992–0.997	< 0.001		0.992	0.987–0.995	< 0.001	
Medial proximal tibial angle	0.879	0.814–0.921	< 0.001		0.904	0.853–0.938	< 0.001	
Lateral distal femoral angle	0.961	0.940–0.975	< 0.001		0.988	0.980–0.992	< 0.001	
Joint-line convergence angle	0.927	0.887–0.952	< 0.001		0.925	0.884–0.951	< 0.001	
K-L grade (FLWAP radiographs)				0.885				0.856
K-L grade (PA radiographs)				0.834				0.854
Group C (*n* = 32)								
Hip-knee-ankle angle	0.996	0.992–0.998	< 0.001		0.999	0.998-1.000	< 0.001	
Weight-bearing line ratio	0.997	0.993–0.998	< 0.001		0.958	0.915–0.980	< 0.001	
Medial proximal tibial angle	0.867	0.727–0.935	< 0.001		0.885	0.765–0.943	< 0.001	
Lateral distal femoral angle	0.927	0.852–0.964	< 0.001		0.985	0.969–0.993	< 0.001	
Joint-line convergence angle	0.901	0.797–0.951	< 0.001		0.893	0.781–0.948	< 0.001	
K-L grade (FLWAP radiographs)				0.751				0.841
K-L grade (PA radiographs)				0.823				0.832

*ICC* Intra-class correlation coefficients, *CI* Confidence interval, *K-L* Kellgren-Lawrence, *FLWAP* full-length weight-bearing anteroposterior, *PA* posteroanterior

## Discussion

The principal finding of the present study was that PTS could affect the measurement reliability regarding the radiographic parameters of the knee. This study revealed that as PTS increases, the measurement reliability of radiographic variables, including parameters related to the joint line such as MPTA and JLCA, could decrease. Therefore, caution should be exercised when measuring or interpreting knee-related radiographic parameters in patients with a high PTS. This study can serve as a reference when performing measurements of knee-related radiographic parameters.

PTS, which directly reflects the bony morphology of the tibia articular surface, is known to exhibit significant inter-individual variability [12–17]. This variability in PTS is reported to have a substantial impact on joint biomechanics [26, 28–30]; on the other hand, it may also influence the measurement accuracy of related radiographic parameters. In practice, there are instances where knee joint lines appear flattened in plain radiographs, while in others, they appear somewhat indistinct making it challenging to precisely determine the articular surface. When faced with difficulties in accurately designating reference points for the joint line, it can in turn affect the precision of measuring radiographic parameters that include the joint line. However, there is a lack of prior research analyzing the impact of PTS on the accuracy of radiographic variable measurements of the knee. Therefore, in this study, we conducted a comparative evaluation of the measurement reliability of radiographic parameters of the knee according to PTS.

**Fig. 4:**
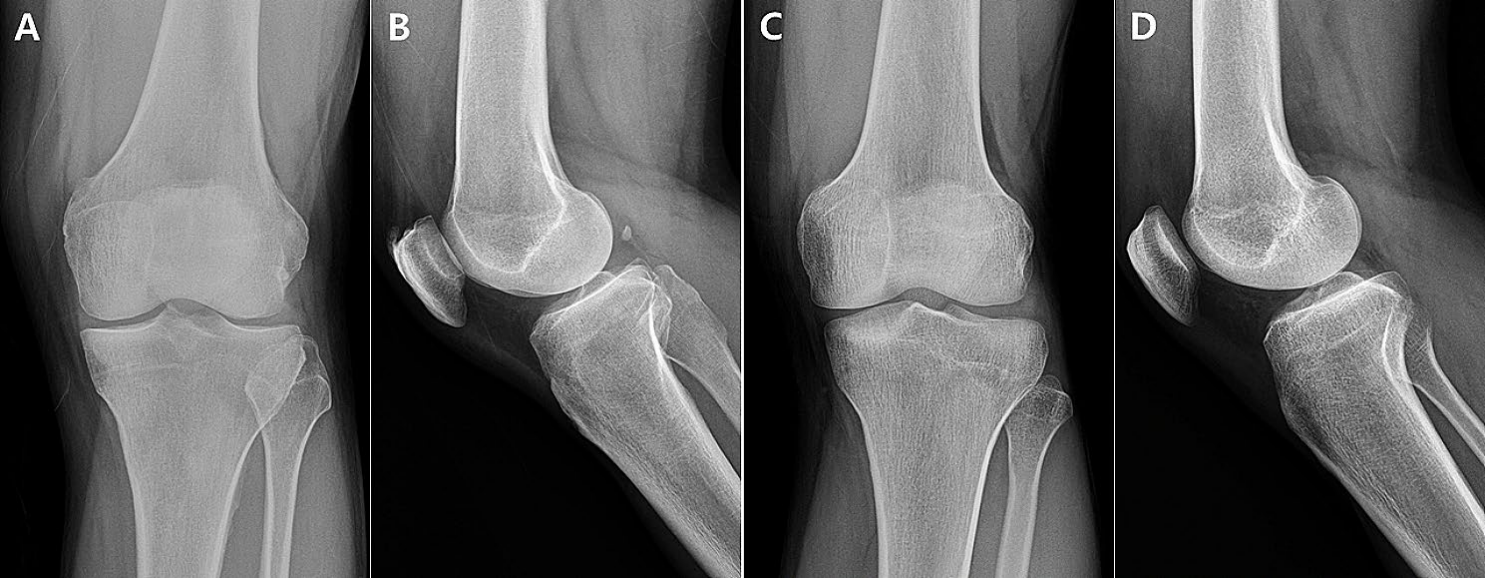
(**A, B**) Patient with a relatively low posterior tibial slope (2.2°) showing distinct joint line. (**C, D**) Patient with a relatively high posterior tibial slope (8.2°) showing indistinct joint line

As hypothesized, PTS was observed to influence the measurement accuracy of radiographic parameters that include knee joint line, such as MPTA and JLCA. Higher PTS resulted in decreased measurement reliability for these variables, consistently observed in both intra-observer and inter-observer reliability assessments. The differences in measurement reliability are considered to be attributable to variations in the radiographic morphology of the tibia articular surface in plain radiographs based on the degree of PTS. While there is a lack of prior related research to support these observations, within the cohort of patients included in this study, individuals with relatively small PTS displayed a distinct and well-defined tibial articular surface characterized by a distinct joint line, whereas those with larger PTS exhibited a comparatively less distinct tendency (Fig. 4). If the joint line appears unclear, it will be difficult to specify reference points for measuring radiographic parameters, resulting in lower measurement accuracy. In this way, the morphology of the tibial articular surface observed on the AP image, represented as the clarity of the joint line, can affect the measurement accuracy of the related radiographic parameters. The morphology of the tibia articular surface in plain radiographs is primarily influenced by the X-ray beam angle during image acquisition and the knee flexion angle or position of the patient. However, in this study, the analysis was based on anterior-posterior radiographs obtained under strictly controlled conditions, and images at risk of being influenced by knee flexion angle or position were excluded from the evaluation beforehand to minimize potential bias. Although it is not possible to entirely rule out the potential impact of these external factors, this study demonstrated that PTS itself can affect the radiographic morphology of the tibia articular surface in anterior-posterior radiographs, specifically the shape of the knee joint line, through a comparative evaluation among 3 groups. Accordingly, when measuring radiographic parameters including the knee joint line, interpretation considering the influence of PTS would be required.

Several limitations exist in the present study. First, this is a retrospective study, which may be at risk of being associated with selection and confounding biases. Second, the analysis in this study was based on a total of only four measurements, 2 times each by 2 observers, which could limit the generalizability of the findings. Third, the presence of knee contracture was confirmed through medical records, and information regarding the knee flexion angle at the time of radiographic image acquisition could not be obtained due to the absence of standing lateral long bone radiographs. Fourth, there are no related preceding studies or specific references for the criteria used to distinguish groups in this study. Therefore, patients were inevitably classified into 3 groups based on the magnitude of PTS values, with the objective of assessing the trend in measurement reliability according to PTS. Finally, in addition to PTS, there may be other factors that may affect the reliability of measurements of radiographic parameters of the knee. While attempts were made to manage these confounding factors to a certain extent during the patient inclusion process, all conditions potentially associated with measurement reliability might not have been controlled for.

Radiographic assessment is an essential factor in knee-related clinical care, and to enhance the quality of this, it is necessary to maximize the accuracy of radiographic evaluations. Even minor errors in radiographic measurements can potentially impact clinical outcomes in various ways. Treatment strategies, including surgical options, may vary based on the results of radiographic measurements, and similarly, the assessment of the following outcomes is also influenced accordingly. In this context, when evaluating radiographic parameters that include knee joint line, such as MPTA and JLCA, efforts are required to consider the influence of PTS. While PTS is not the sole factor influencing the measurement reliability of these radiographic parameters, a high PTS may serve as a kind of cautionary signal when measuring knee-related radiographic parameters. For patients with a relatively high PTS, to minimize possible measurement errors, careful measurement along with the repetitive measurements for verification may be required. The findings of this study will contribute to reducing unnecessary errors in the measurement of radiographic parameters associated with the knee joint.

## Conclusion

The larger the PTS, the lower the measurement reliability regarding the radiographic parameters of the knee that includes the joint line, such as MPTA and JLCA. Given the occasional challenge in accurately identifying the knee joint line in patients with a relatively large PTS, careful measurement of radiographic parameters is crucial and utilizing repetitive measurements for verification may contribute to minimizing measurement errors.

### Electronic supplementary material

Below is the link to the electronic supplementary material.

Supplementary Material 1

Supplementary Material 2

Supplementary Material 3

Supplementary Material 4

## Data Availability

The datasets used and/or analyzed in this study available from the corresponding author on reasonable request.

## Abbreviations

AP: Anteroposterior
PTS: Posterior tibial slope
FLWAP: Full-length weight-bearing anteroposterior
PA: Posteroanterior
HKA: Hip-knee-ankle
WBLR: Weight-bearing line ratio
MPTA: Medial proximal tibial angle
LDFA: Lateral distal femoral angle
JLCA: Joint-line convergence angle
ICC: Intra-class correlation coefficients
