# Supplementary material for: The posterior tibial slope affects the measurement reliability regarding the radiographic parameter of the knee
Source: BMC Musculoskelet Disord. 2024 Mar 7;25:202. doi: 10.1186/s12891-024-07330-3 (PMC10918909; doi:10.1186/s12891-024-07330-3)
Supplement: Supplementary file 1 — Supplementary Material 1 [file 12891_2024_7330_MOESM1_ESM.docx]

**Supplementary material 1.** Measurement reliabilities for the posterior tibial slope.

| Variables | ICC | 95% CI |
| --- | --- | --- |
| Intra-observer reliabilities |  |  |
| Observer 1-Measurement 1 / Observer 1-Measurement 2 | 0.990 | 0.986-0.992 |
| Observer 2-Measurement 1 / Observer 2-Measurement 2 | 0.982 | 0.975-0.987 |
| Inter-observer reliabilities |  |  |
| Observer 1- Measurement 1 / Observer 2- Measurement 1 | 0.970 | 0.960-0.978 |
| Observer 1- Measurement 2 / Observer 2- Measurement 2 | 0.959 | 0.945-0.970 |
| Observer 1- Measurement 1 / Observer 2- Measurement 2 | 0.969 | 0.959-0.977 |
| Observer 1- Measurement 2 / Observer 2- Measurement 1 | 0.955 | 0.940-0.967 |

*ICC* Intra-class correlation coefficients, *CI* Confidence interval
