# Supplementary figures and images for: The posterior tibial slope affects the measurement reliability regarding the radiographic parameter of the knee
Source: BMC Musculoskelet Disord. 2024 Mar 7;25:202. doi: 10.1186/s12891-024-07330-3 (PMC10918909; doi:10.1186/s12891-024-07330-3)

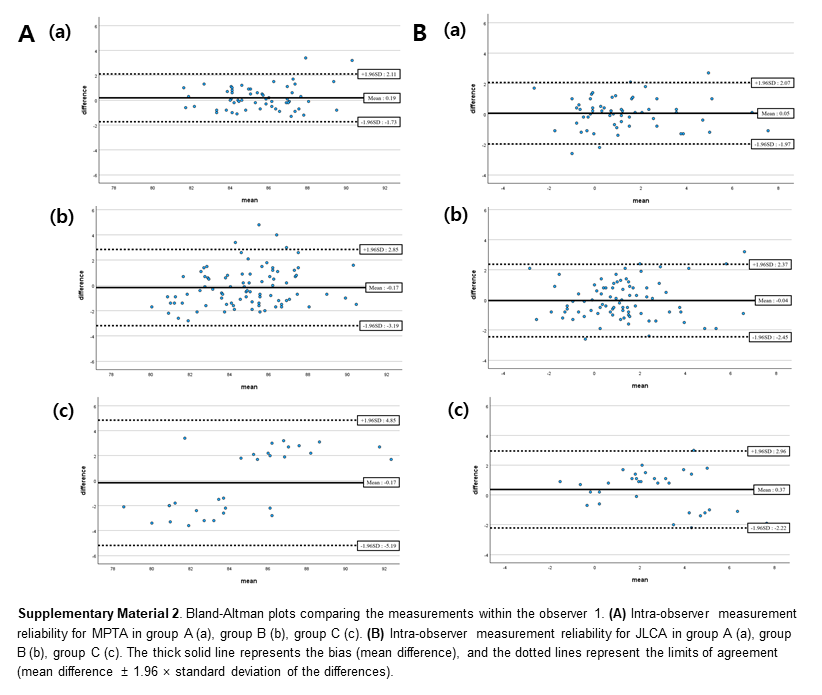

Supplement: Supplementary file 2 — Supplementary Material 2 [file 12891_2024_7330_MOESM2_ESM.png]

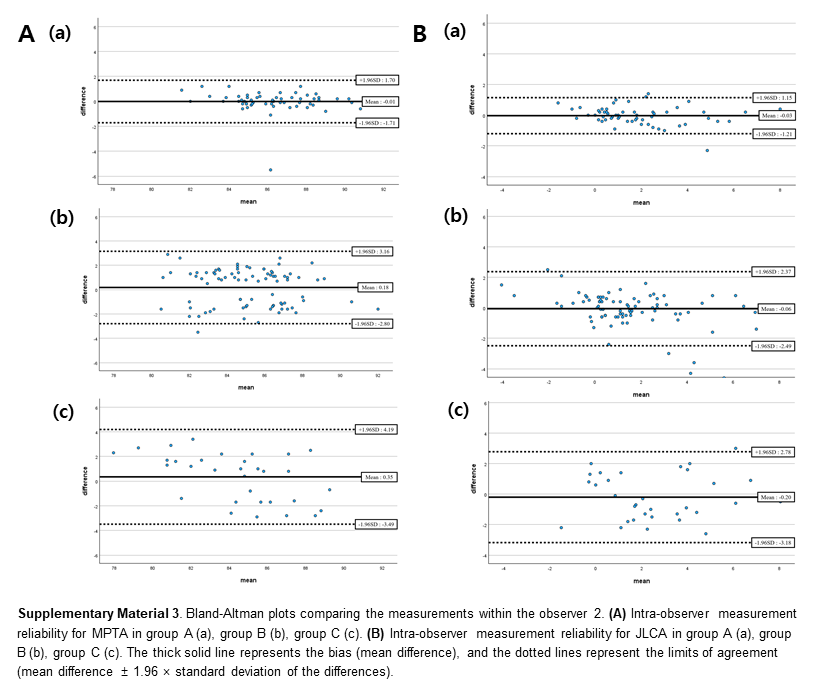

Supplement: Supplementary file 3 — Supplementary Material 3 [file 12891_2024_7330_MOESM3_ESM.png]

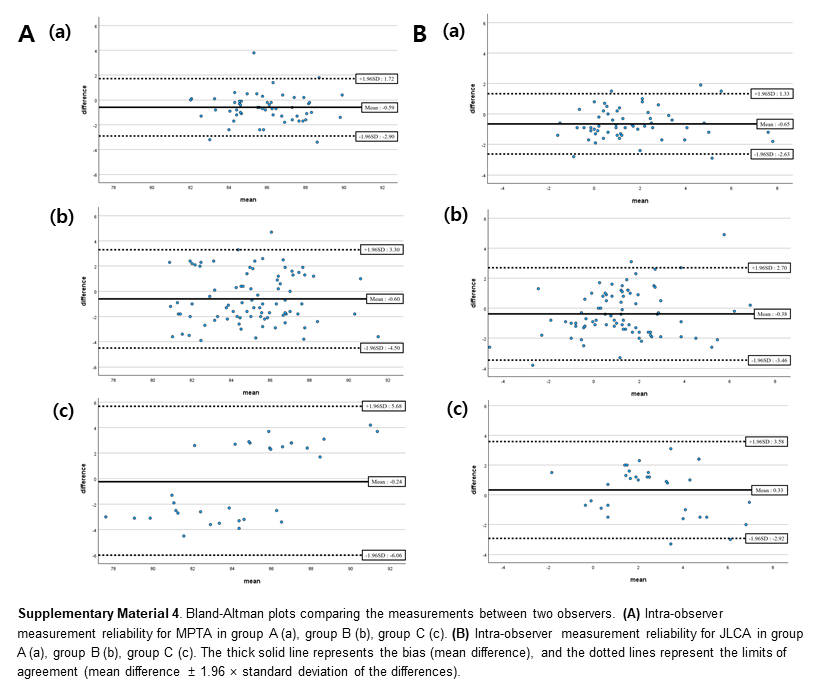

Supplement: Supplementary file 4 — Supplementary Material 4 [file 12891_2024_7330_MOESM4_ESM.png]
